# Brown Seaweed Phlorotannins: Chemical Diversity, Sustainable Extraction, Selective Quantification, Ageing-Related Bioactivities, and Phlorotannin-First Biorefinery Potential

**DOI:** 10.3390/md24080259

**Published:** 2026-07-26

**Authors:** Liaqat Zeb, Monica Jordheim

**Affiliations:** Department of Chemistry, University of Bergen, 5007 Bergen, Norway; liaqat.zeb@uib.no

**Keywords:** brown seaweed, phlorotannins, NADES, qNMR, ageing, antioxidant, extraction, biorefinery

## Abstract

Phlorotannins are the characteristic phenolic metabolites of brown seaweeds and represent one of the most chemically diverse, analytically challenging, and industrially promising classes of marine polyphenols. Unlike terrestrial phenolics, phlorotannins are formed from phloroglucinol units and occur as complex mixtures of fucols, phlorethols, fuhalols, fucophlorethols, eckols, and related oligomeric or polymeric structures whose composition varies strongly with species, tissue, season, habitat, extraction protocol, and purification strategy. This review critically examines brown seaweed phlorotannins across five major dimensions: chemical diversity and species-dependent availability; extraction, enrichment, and sustainable solvent strategies, including natural deep eutectic solvents (NADES); analytical challenges and advances in selective quantification, highlighting the non-specific nature of Folin–Ciocalteu assays and the emerging value of selective qNMR-based quantification; and biological relevance to ageing-associated processes such as oxidative stress, inflammation, glycation, metabolic dysfunction, neurodegeneration, and skin ageing. The review further evaluates translational barriers, including poor standardization, uncertain bioavailability, matrix interference, limited compound-specific standards, and insufficient in vivo and clinical validation. Finally, a phlorotannin-first brown seaweed biorefinery framework is proposed, in which the phenolic fraction is recovered early as a high-value stream before sequential valorization of polysaccharides, proteins, pigments, minerals, and residual biomass. This integrated perspective highlights the need to move from crude “total phenolic” descriptions toward chemically defined, functionally validated, and industrially scalable phlorotannin systems.

## 1. Introduction

**Figure 1:**
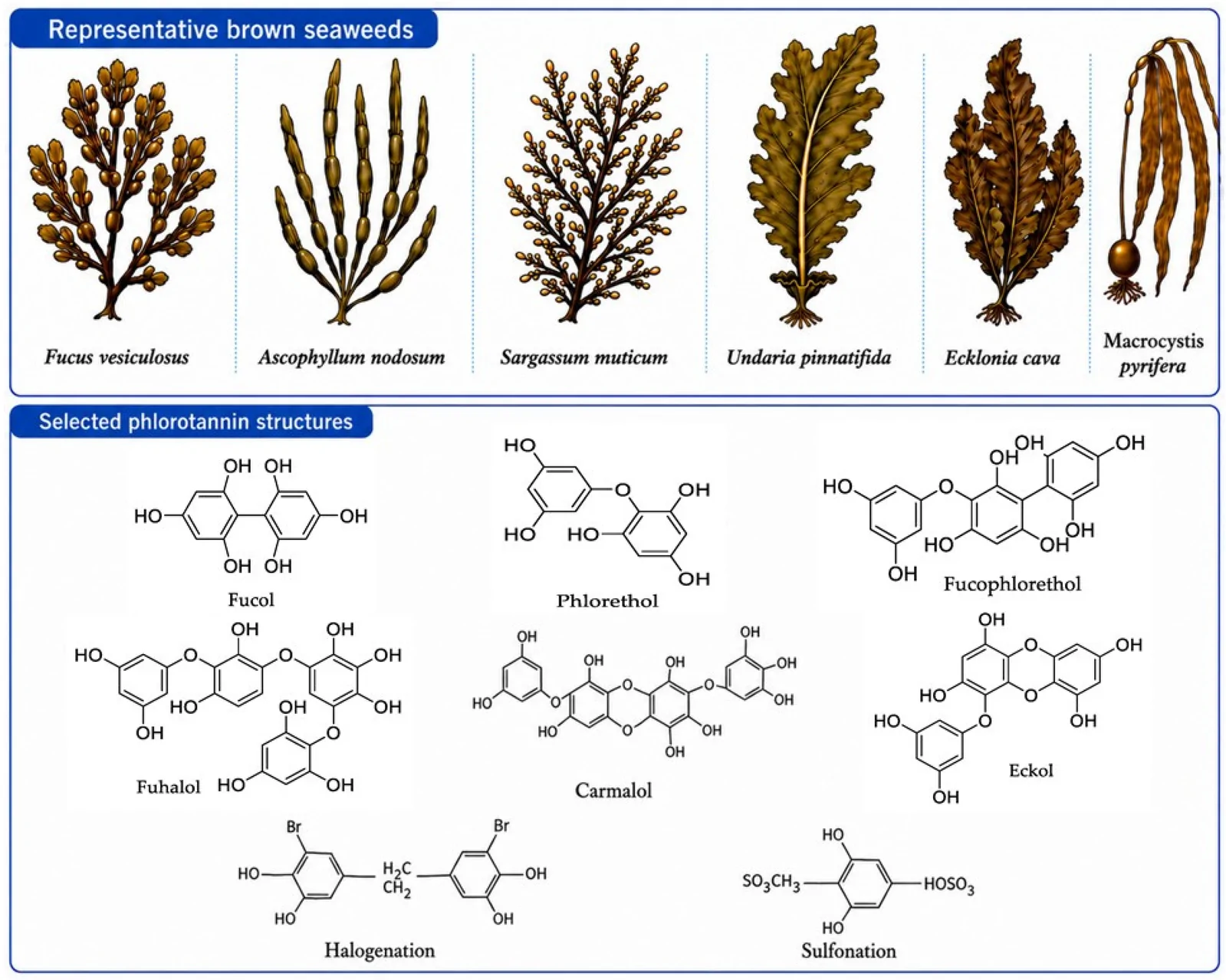
Representative brown seaweed sources and major structural classes of phlorotannins.

Brown seaweeds constitute one of the most chemically distinctive groups of marine biomass because they combine ecological abundance with a complex metabolite collection that includes polysaccharides, pigments, minerals, lipids, and the brown-algal polyphenols known as phlorotannins [1,2,3,4,5,6,7,8]. Among these constituents, phlorotannins represent the defining phenolic signature of Phaeophyceae and are increasingly viewed as high-value marine metabolites for food, nutraceutical, pharmaceutical, cosmeceutical, and biorefinery applications [3,4,5,9,10]. Phlorotannins are structurally specialized polymers of phloroglucinol units linked mainly through aryl–aryl and/or aryl–ether bonds, producing oligomeric and polymeric structures [3,5,6,11,12,13,14]. This structural diversity gives rise to several recognized subclasses, including fucols, phlorethols, fuhalols, fucophlorethols, and eckol-type compounds (Figure 1) [7,11,14,15,16]. Consequently, the term “phlorotannin-rich extract” should not be interpreted as a chemically uniform entity, because extracts from different species, solvents, seasons, tissues, and purification workflows can differ substantially in composition and bioactivity [10,12,17,18,19,20]. The biological interest in phlorotannins has expanded from classical antioxidant screening to a broader spectrum of activities, including anti-inflammatory, anti-microbial, anti-viral, anti-diabetic, anti-obesity, anticancer, anti-glycation, neuroprotective, photoprotective, anti-senescence, and cosmeceutical effects [9,20,21,22,23,24,25,26,27,28,29,30,31,32]. These activities are particularly relevant to ageing-related biology because oxidative stress, chronic low-grade inflammation, impaired glucose regulation, carbonyl stress, mitochondrial dysfunction, and extracellular-matrix degradation are central features of many age-associated disorders [2,4,23,33,34]. However, most reported effects remain based on in vitro systems, enriched fractions, or early preclinical models, and stronger translational evidence is still required before robust health or therapeutic claims can be made [1,7,9,35,36,37,38,39,40,41].

Understanding the ecological function of phlorotannins helps explain their variability, as these compounds are endogenous algal metabolites involved in defense, cell-wall structure, oxidative-stress protection, ultraviolet tolerance, and microbial interactions [1,6,42,43,44,45,46,47]. Their abundance and qualitative profile are influenced by species identity, life stage, tissue type, season, locality, habitat exposure, nutrient conditions, and light regime, making brown seaweed biomass chemically dynamic rather than compositionally fixed [5,7,12,40,45,46]. Such ecological flexibility directly influences industrial and biomedical applications, since harvest timing, site selection, and tissue choice can alter extraction yield, fraction composition, and apparent bioactivity before laboratory processing begins [15,19,48,49]. The limitations of FC assays are discussed in Section 4.1. Advanced analytical methods are increasingly used for phlorotannin characterization and quantification (Section 4).

Extraction science is equally central because the composition and bioactivity of a phlorotannin fraction are shaped by solvent polarity, extraction time, temperature, liquid-to-solid ratio, biomass pretreatment, oxidation state, and downstream purification [10,17,19,32,49,50,51,52,53,54]. Conventional solid–liquid extraction with hydro-organic solvents remains foundational, but intensified methods are gaining attention because they may improve mass transfer, selectivity, processing time, and sustainability [48,49,55,56]. NADES are particularly promising to recover polyphenol/phlorotannins while reducing reliance on volatile petroleum-derived solvents [17,50,51,55,56,57]. Purification is indispensable because crude seaweed extracts frequently contain co-extracted compounds that may contribute to observed effects [3,13,58,59,60]. Antioxidant, anti-inflammatory, antidiabetic, and anti-glycation responses can differ markedly between crude extracts and purified fractions, making extraction and purification history essential for study interpretation [16,23,27,30,41,61]. The translational impact of phlorotannins is strongly influenced by bioavailability and gastrointestinal fate (Section 5.1). Therefore, this review links species-dependent chemistry, sustainable extraction, selective quantification, ageing-related mechanisms, translational barriers, and a phlorotannin-first biorefinery model within a single critical framework. Literature searches were conducted during 2025 and updated during manuscript revision to incorporate newly published studies relevant to phlorotannin research. Consequently, a limited number of references published in 2026 are included to provide the most current perspective available at the time of manuscript preparation.

## 2. Chemical Foundation, Biosynthesis, and Comparative Species Availability of Phlorotannins

Phlorotannins can be classified into subclasses such as fuhalols and phlorethols (phlorotannins with an ether linkage), fucols (with a phenyl linkage), fucophloroethols (with an ether and phenyl linkage), and eckols (with a dibenzodioxin linkage) [1,3,5,6,7,11,13,14,42,62]. Phlorotannins are a structurally distinctive class of brown-algal polyphenols, biosynthesized primarily in Phaeophyceae as dehydro-polymers of phloroglucinol, or 1,3,5-trihydroxybenzene, through the acetate–malonate/polyketide pathway [1,3,7]. Their molecular architecture arises from repeated coupling of phloroglucinol units through aryl–aryl and/or aryl–ether bonds, generating linear, branched, cyclic, and highly polymerized structures with broad variation in molecular mass, oxidation state, polarity, and physicochemical behavior [1,6,7,63].

The most useful structural classification is based on inter-phloroglucinol linkage type: fucols contain C–C aryl linkages, phlorethols contain aryl–ether linkages, fuhalols are oxygen-rich ether-linked analogues, fucophlorethols contain both C–C and C–O–C linkages, and eckol- or carmalol-type compounds contain dibenzodioxin-like frameworks [3,11,13].

Linkage pattern and hydroxylation influence solubility, oxidation behavior, chromatographic retention, protein and polysaccharide binding, membrane interactions, mass-spectrometric fragmentation, and likely bioavailability, making this classification chemically important [1,6,7]. Natural phlorotannin extracts usually contain complex mixtures rather than single purified compounds, and extensive isomerism, limited commercial standards, and overlapping spectroscopic behavior make complete structural assignment difficult using any single analytical platform [2,3,12,13,64,65,66].

Comparative species analysis is necessary because brown seaweeds differ substantially in both total phlorotannin richness and dominant structural classes [28,40,47,60,66,67,68,69,70,71,72]. For example, *A. nodosum* is a strategically important model because it combines North Atlantic abundance, industrial availability, Fucales-type phlorotannin chemistry, and strong relevance to antioxidant and antidiabetic screening [23,26,27,33,40,45,46,60,73,74,75,76,77,78,79,80,81]. *F. vesiculosus* is another benchmark species because it is widely used in studies of Fucaceae phlorotannin composition, fractionation, antioxidant behavior, anti-glycation activity, gut-fate questions, and green extraction development [15,16,17,30,37,42,51,53,64,82,83,84,85,86,87,88,89,90,91]. *Ecklonia* species are especially important sources of eckol, dieckol, and phlorofucofuroeckol-type compounds and are therefore strongly represented in mechanistic pharmacology and cosmeceutical research (Table 1) [20,22,29,41,47,92,93,94,95,96,97,98,99,100,101,102,103,104,105,106]. Saccharina, Sargassum, Macrocystis, and other brown algal taxa broaden the comparative landscape by showing that lower total phenolic richness does not necessarily imply low technological relevance, because matrix effects, extractability, cultivation potential, and biorefinery compatibility also determine value [10,12,18,19,34,35,50,58,65,69,70,72,76,78,107,108,109,110,111,112,113,114,115,116,117,118,119,120,121,122,123,124,125].

Integrating the chemical foundation of phlorotannin research rests on four connected principles: phloroglucinol-derived biosynthesis, linkage-based structural diversity, ecophysiological regulation, and strong species-dependent availability [1,7,66]. These principles are essential for interpreting extraction efficiency, analytical data, antioxidant or enzyme-inhibitory readouts, bioavailability claims, and translational potential across different brown seaweed taxa [2,3,7,14,66,68]. For mechanistic extraction research, Fucales species such as *Ascophyllum* and *Fucus* remain ideal because their phenolic signals are strong enough to stress-test solvent systems and quantification platforms [1,2,15,16,45,79,81,84]. For compound-level pharmacology, *Ecklonia* remains indispensable because its eckol-centered chemistry is relatively tractable and biologically well explored [22,29,47,92,93,94,95]. For industrially realistic scaling and coupling to hydrocolloid value chains, *Saccharina* and *Undaria* are highly relevant [10,50,58,76,78,106,107,108,126,127,128,129]. For the valorization of underused biomass, *Sargassum* and *Macrocystis* offer engineering and sustainability opportunities [109,110,111,124,125]. Species selection should be guided by the analytical, biological, or industrial objective, with clear justification of why a particular taxon was chosen. This would improve comparability among studies and strengthen translational relevance. That methodological discipline would substantially improve comparability and would help the field move from descriptive enthusiasm to reproducible translational phlorotannin science.

**Figure 2:**
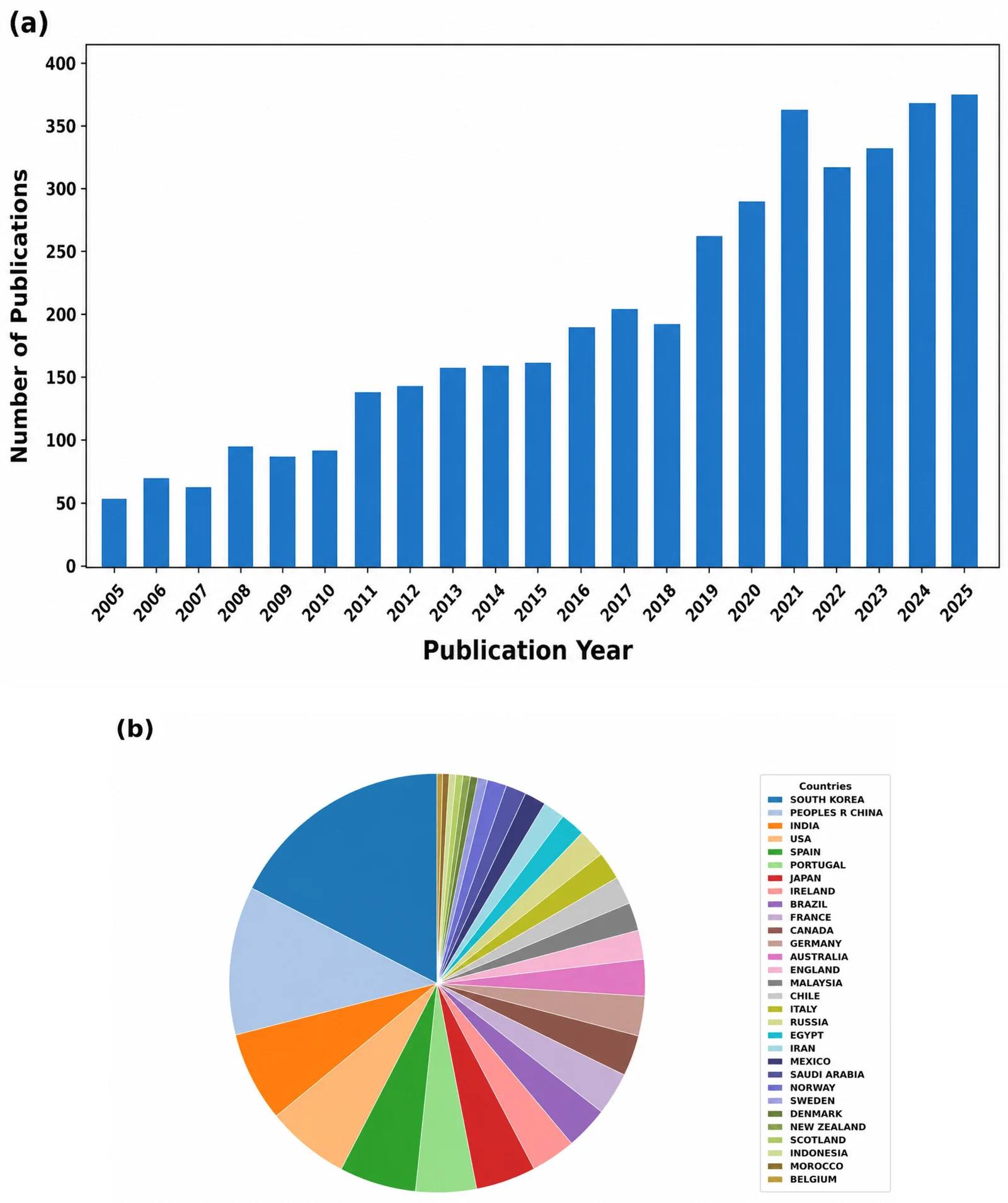

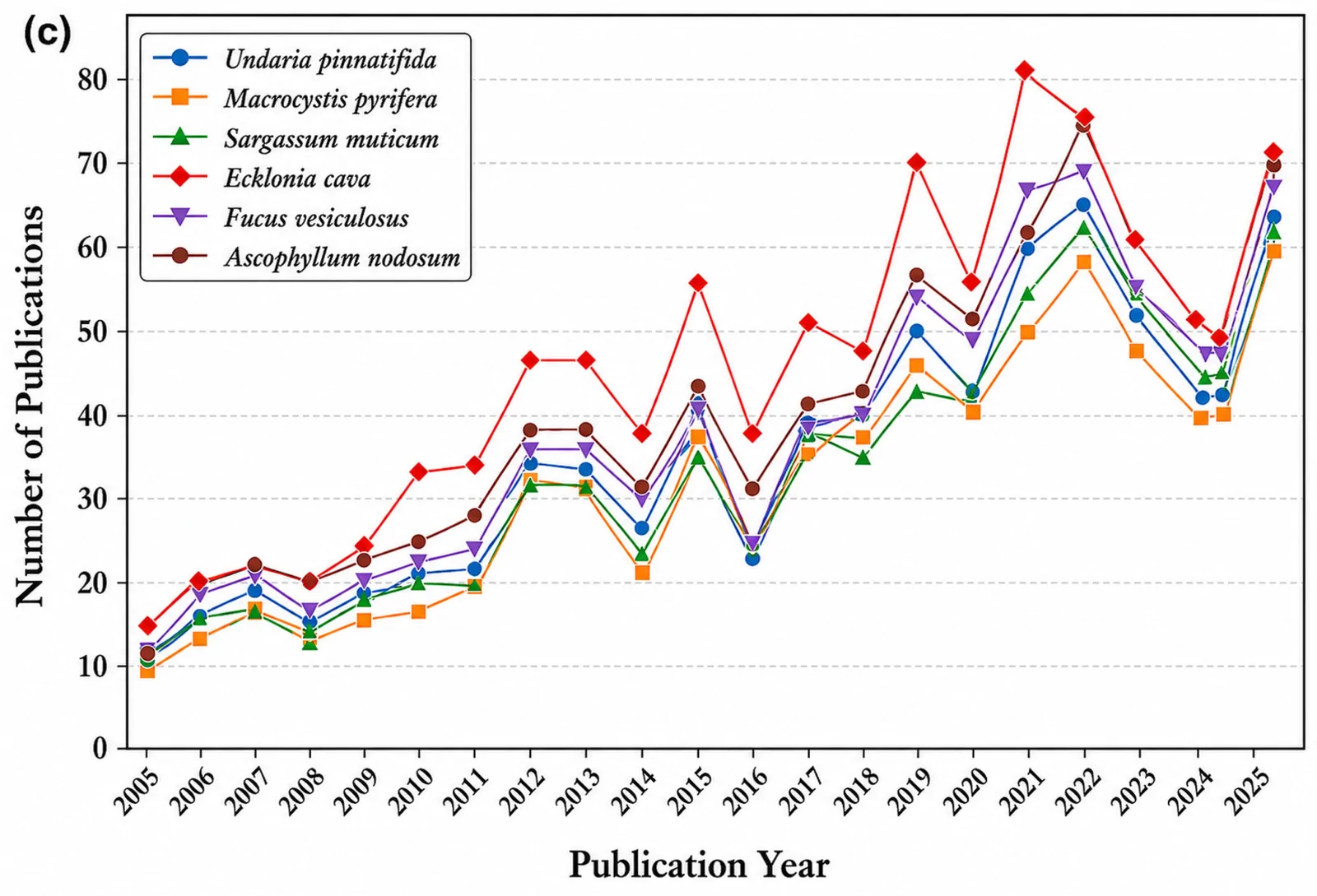
(**a**) Global publication trends in brown seaweed phlorotannin research from 2005 to 2025 based on Web of Science data. (**b**) Country-level contributions to brown seaweed phlorotannin research from 2005 to 2025 based on Web of Science data. (**c**) Publication trends for major brown seaweed phlorotannin sources from 2005 to 2025. The brown seaweed species *Undaria pinnatifida*, *Macrocystis pyrifera*, *Sargassum muticum*, *Ecklonia cava*, *Fucus vesiculosus*, and *Ascophyllum nodosum* were searched using their complete species names in combination with “phlorotannin” or “phlorotannins”.

Figure 2a,b shows the growth of brown seaweed phlorotannin research from 2005 to 2025, with publications increasing strongly after 2011. Research activity appears concentrated in countries with strong marine biotechnology and seaweed research programmes, including South Korea, China, India, the USA, Spain, Portugal, Japan, Ireland, Brazil, and France (Figure 2b). The species trend also shows that *Ascophyllum nodosum*, *Fucus vesiculosus*, and *Ecklonia cava* are the most studied genera (Figure 2c). This reflects their availability, chemical richness, and relevance for bioactivity studies. Table 1 confirms strong species-level variation in phlorotannin composition. *A. nodosum* is mainly associated with fucol, phlorethol, and fucophlorethol-type compounds. *F. vesiculosus* shows broader diversity, including hexafucol, tetrafucophlorethol, fucofuropentaphlorethol, heptafucol, trifucotriphlorethol, difucotetraphlorethol, pentafucodiphlorethol, hexafucophlorethol, and tetrafucotetraphlorethol. Other species, including *Macrocystis pyrifera*, *Laminaria digitata*, *Undaria pinnatifida*, and *Eisenia arborea*, expand the chemical landscape (Figure 2 and Table 1).

**Table 1:** Species-level diversity of reported phlorotannin oligomers and compound groups in brown seaweeds.

Species	Reported Phlorotannin Oligomers/Compound Groups	References
*Ascophyllum nodosum*	Fucol/phlorethol/fucophlorethol	[23,26,27,33,40,45,46,60,75,76,78,130]
*Bifurcaria bifurcata*	Trihydroxyheptafuhalol	[131]
*Carpophyllum flexuosum*/*Cystophora flexuosum*	Hydroxyhexafuhalol; dihydroxyhexafuhalol; bifuhalol; hydroxytrifuhalol;tetrafuhalol	[68,106]
*Cystoseira abies-marina*	Fucol/phlorethol/fucophlorethol	[132]
*Cystoseira barbata*	Fucol/phlorethol/fucophlorethol	[133]
*Durvillaea antarctica*	Fucol/phlorethol/fucophlorethol	[134]
*Fucus distichus*	Fucol/phlorethol/fucophlorethol	[135]
*Fucus spiralis*	Fucol/phlorethol/fucophlorethol	[64,136,137,138]
*Fucus vesiculosus*	Fucol/phlorethol/fucophlorethol; hexafucol; tetrafucophlorethol; fucofuropentaphlorethol; heptafucol; trifucotriphlorethol; difucotetraphlorethol; pentafucodiphlorethol; hexafucophlorethol; tetrafucotetraphlorethol	[15,16,17,30,42,51,53,64,82,85,86,89,90,131,139]
*Sargassum fusiforme*	Fucol/phlorethol/fucophlorethol; tetraphlorethoxycarmalol; hexafuhalol; hydroxyhexafuhalol; dihydroxytetraphlorethoxycarmalol; dihydroxyhexafuhalol; pentaphlorethoxycarmalol; heptafuhalol; hydroxyheptafuhalol; dihydroxypentaphlorethoxycarmalol; dihydroxyheptafuhalol; hexaphlorethoxycarmalol; dihydroxynonafuhalol; trihydroxyhexaphlorethoxycarmalol; trihydroxyoctafuhalol; trihydroxynonafuhalol; trihydroxydecafuhalol; tetrahydroxydecafuhalol; tetrahydroxyfuhalol; pentahydroxyfuhalol	[35,69,70,78,116]
*Sargassum muticum*	Hexaphlorethol; hexafuhalol; hydroxyhexafuhalol; dihydroxyhexafuhalol; trihydroxyhexafuhalol; heptaphlorethol; hydroxyheptafuhalol; dihydroxyheptafuhalol; trihydroxyheptafuhalol; octafuhalol; dihydroxynonafuhalol; trihydroxyoctafuhalol; nonafuhalol; decafuhalol	[110,114,117,119,122]
*Sargassum vulgare*	Dibenzodioxine-1,3,6,8-tetraol; fuhalol; pentaphlorethol; fucopentaphlorethol; dihydroxypentafuhalol	[12]
*Macrocystis pyrifera*	Phloroeckol; tetrameric phlorofucofuroeckol; phloroethol o fucols	[124,125]
*Laminaria digitata*	Fuhalol; carmalol; phloroethol; fucophloroethol	[43,140,141]
*Laminaria hyperborea*	Phlorotannins trimer; tetramer; hexamer; sulfated dimer	[58,142]
*Eisenia arborea*	Eckol; phlorofucofuroeckol; phloroeckol; fucophloroethol; phloroethol o fucols	[143]
*Ecklonia kurome*	Eckol; phlorofucofuroeckol; dieckol; 8,8′-bieckol; phloroethol	[93,100]
*Ecklonia stolonifera*	Phlorofucofuroeckol A; dieckol; dioxinodehydroeckol	[20,47]
*Ecklonia cava*	Dieckol; 8,8′-bieckol; 7-phloroeckol; 2-O-(2,4,5-trihydroxyphenyl)-6,6′-bieckol; 6,6′-bieckol; phlorofucofuroeckol-A; eckol; 2-phloroeckol; fucofuroeckol	[22,29,41,47,92,94,95,96,97,101,104,105]
*Ecklonia radiata*	Fucol;fucodiphlorethol G; phloroeckol; eckol; phloroeckol isomer 2; dieckol; diphlorethohydroxycarmalol; 6,6’-bieckol; eckmaxol; eckmaxol isomer 1; dieckol isomer 1; dioxinodehydroeckol; phlorofucofuroeckol	[99,102,103]
*Undaria pinnatifida*	Bifuhol; trifuhalol; tetrafuhalol	[78,127,128,129]

## 3. Extraction Methods and Sustainable Recovery of Phlorotannins

Phlorotannin production can be divided into upstream and downstream operations. Upstream operations include species selection, breeding or cultivation strategy, site selection, seasonal harvest timing, biomass collection, washing/desalting, drying, milling, moisture control, stabilization, storage, and transport [48,55,56,144,145]. These steps determine the chemical starting material because phlorotannin content and composition vary across species, tissues, seasons, habitat, light exposure, nutrient conditions, and post-harvest handling [4,11,13,66]. Downstream operations begin after stabilized biomass is obtained and include conventional solid–liquid extraction, aqueous ethanol extraction, acetone- or methanol-based analytical extraction, NADES/DES extraction, ultrasound-assisted extraction, microwave-assisted extraction, pressurized-liquid extraction, enzyme-assisted extraction, solid–liquid separation, adsorption-resin purification, membrane concentration, chromatographic fractionation, solvent recovery, drying, formulation, and analytical validation [1,4,56,66,144,145]. Upstream decisions control biomass quality and native phlorotannin availability, whereas downstream decisions determine extraction yield, selectivity, purity, cost, scalability, and regulatory suitability.

The extraction of phlorotannins from brown seaweeds should be regarded as an integrated workflow rather than a simple solvent-transfer process. It involves biomass preparation, solvent-assisted release, selective recovery, preservation of native structures, enrichment or purification, and analytical validation [11,13,63]. The complexity arises from the fact that phlorotannins may occur as soluble intracellular compounds or as cell-wall-associated phenolics, and they are commonly co-extracted with matrices and other reducing substances. Therefore, extraction conditions must be selected according to the intended application, whether rapid screening, preparation of antioxidant-rich crude extracts, production of purified phlorotannin concentrates, structural elucidation, development of food-grade ingredients, or integration into scalable seaweed biorefinery processes [11,66]. The solvent choice, extraction technology, purification strategy, and analytical method strongly influence both yield and chemical interpretation of phlorotannin-rich extracts [13,60,62,116]. Pre-processing steps such as harvesting, washing, desalting, drying, milling, moisture control, and storage are critical because phlorotannins are chemically sensitive and their recovery can be affected by biomass condition before extraction begins [59]. Conventional solid–liquid extraction remains the most widely used baseline method and commonly employs water, ethanol, methanol, acetone, or hydro-organic mixtures, with solvent polarity, temperature, extraction time, solvent-to-solid ratio, and particle size controlling both yield and selectivity [13,49,56,119,146]. Ethanol–water systems are especially attractive for food, nutraceutical, and cosmeceutical applications because ethanol is more suitable for downstream use than methanol or acetone, although analytical studies may still use stronger organic solvents for recovery and profiling [56,63]. In a study of *Sargassum fusiforme*, the authors identified 30% ethanol–water as the optimal solvent system, with a solid/liquid ratio of 1:5, extraction temperature of 25 °C, and extraction time of 30 min, yielding 63.35–63.61 mg PGE g^−1^ dry biomass under the optimized conditions [116]. Extraction efficiency increased from 10% to 30% ethanol but decreased at higher ethanol concentrations [116]. The study also found that room-temperature extraction outperformed higher-temperature extraction for this matrix, suggesting that thermolability and oxidation can outweigh the usual mass-transfer benefits of heat and against overgeneralizing extraction conditions across species [116]. A major limitation of conventional extraction is that maximizing total phenolic yield does not necessarily maximize phlorotannin selectivity or biological relevance [13,146]. Additional purification may be required depending on the intended analytical or biological application [48,56,102]. Therefore, crude extracts often require liquid–liquid partitioning, adsorption resins, membrane concentration, Sephadex LH-20 fractionation, preparative chromatography, or other enrichment strategies before reliable structure–activity relationships can be proposed [48,58,142]. Advanced analytical methods are increasingly used to improve the characterization and quantification of phlorotannins, as discussed in Section 4.

Advances in phlorotannin extraction have shifted the field from yield-oriented solvent extraction toward greener, faster, more selective, and application-specific workflows [6,11,117]. Ultrasound-assisted extraction (UAE) improves solvent penetration and mass transfer through cavitation, microstreaming, and matrix disruption, while microwave-assisted extraction (MAE) accelerates release through rapid dielectric heating of hydrated algal tissues (Table 2) [48,50,53,70]. In *A. nodosum*, ultrasound has been reported to enhance the extraction of high-molecular-weight phenolic compounds and to improve solute diffusion and dissolution from the seaweed surface. The main advantages of the UAE are shorter extraction times, lower solvent demand than some batch processes, and good compatibility with aqueous ethanol. Ultrasound enhanced recovery under mild conditions [26,81].

Microwave-assisted extraction of *F. vesiculosus* using hydroethanolic solvent systems illustrates how process intensification can improve phlorotannin recovery, although extraction power, temperature, solvent composition, and time must be carefully optimized to avoid degradation of thermolabile compounds [53]. In mechanistic terms, microwaves heat the matrix internally, promote ionic migration, and increase cell wall porosity, thereby facilitating the release of target compounds. Nevertheless, the study warns that both MAE and pressurized liquid extraction (PLE) may partially degrade thermolabile compounds at excessive temperatures [13,53,67,146]. This is particularly relevant for phlorotannins, whose oxidation state and polymeric complexity can be altered during aggressive treatment. For this reason, MAE should be considered a high-efficiency option for controlled extraction, not a justification for indiscriminate thermal loading [11,28,66].

Pressurized liquid extraction, enzyme-assisted extraction, pulsed electric field-assisted extraction, hydrodynamic cavitation, and other emerging technologies are also being investigated because they can improve cell-wall disruption, reduce solvent use, shorten extraction time, and support industrially scalable recovery of brown-algal bioactives [13,18,40,48,56,80,89,107,125,132,147,148]. PLE uses elevated pressure and temperature to enhance solvent penetration, accelerate desorption, and reduce solvent volume and extraction time. PLE typically operates at up to 15 MPa and 200 °C and can achieve higher productivity than traditional extraction, although partial degradation of thermolabile compounds remains a concern [66,89]. PLE outperformed traditional solid–liquid extraction for phenolic recovery from Irish macroalgae, including *A. nodosum*, *Pelvetia canaliculata*, and *Fucus spiralis* [147]. PLE is particularly relevant for industrial translation because it reduces extraction time and solvent volume, but its use requires careful balancing of efficiency against selectivity and chemical preservation. Where the end goal is a food-grade antioxidant fraction, moderate conditions may be preferable. Where the goal is maximal phenolic capture for subsequent purification, more extensive conditions may be tolerated if degradation is monitored analytically [11,13,66,89,146,149].

Enzyme-assisted extraction (EAE) is especially valuable when processing brown algae because the cell wall is complex and resistant, and enzymatic pretreatment can selectively weaken structural barriers under comparatively mild conditions [6,56,150]. An ultrasonic-assisted enzymatic extraction (UAEE) protocol significantly enhanced phlorotannin recovery from *Sargassum horneri*, achieving a total phenolic content (TPC) of 21.51 mg PGE/g DW. Subsequent purification using solvent extraction and Sephadex LH-20 yielded high-purity alginate phlorotannins (SHP), exhibiting potent DPPH radical-scavenging and tyrosinase-inhibitory activities, with IC_50_ values of 22.71 and 26.90 μg/mL, respectively [150]. Structural elucidation based on NMR and MS data suggested that SHP predominantly comprises fucophloroethols, with a degree of polymerization ranging from 3 to 8. Reciprocal plot analysis indicated that SHP inhibits tyrosinase activity through a competitive inhibition mechanism, while fluorescence spectroscopy revealed a dynamic quenching effect of SHP on tyrosinase [150]. EAE is especially valuable where phlorotannins are strongly associated with cell-wall components or where gentle processing is needed to preserve thermolabile molecules, but limitations are also clear: enzyme costs, substrate specificity, incomplete release of all phenolic pools, and the need to carefully optimize reaction time to avoid unnecessary processing [13,56,66,150].

Supercritical CO_2_ extraction and CO_2_-based co-solvent systems have also been applied to brown seaweeds. Supercritical extraction is environmentally attractive because carbon dioxide is non-flammable and readily removable. However, an important limitation is that phlorotannins are relatively polar, so pure CO_2_ is usually inadequate without polar modifiers such as ethanol [14,122,128,151]. *Sargassum muticum, Undaria pinnatifida*, and other species have been explored in this context, and yields can increase when the co-solvent polarity is appropriately tuned [122,128]. For phlorotannins specifically, SFE is therefore best regarded as a niche or hybrid method rather than the default platform. It may fit well in integrated processes aimed at simultaneous recovery of lipophilic and phenolic fractions, but it is less straightforward than hydro-organic extraction for polar polyphenols [3,7,56,66,128,151]. Phlorotannin extraction is progressing from conventional solvent recovery toward sustainable extraction strategies and industrial applications [11,51,56,57,66,70]. The integration of advanced methods and comparison with conventional methods will advance the extraction sciences [55,56].

### Advancement in the Sustainable Extraction of Phlorotannins from Brown Seaweed Using NADES

NADES are liquid systems formed by combining naturally derived hydrogen-bond donors and acceptors in defined molar ratios. Their relevance for algal extraction arises from their tunable polarity, hydrogen-bonding capacity, viscosity, and water tolerance, which can be adjusted to enhance bioactive recovery while reducing reliance on conventional organic solvents [55,56,152]. However, extraction performance remains strongly dependent on algae species, solvent composition, water content, extraction conditions, and the analytical method used to recover algal bioactives. NADES have been used to recover polyphenols andphlorotannins from different brown seaweed species, as indicated in Table 2. For example, Obluchinskaya et al. (2019) evaluated NADES based on choline chloride, lactic acid, betaine, and glucose for *Fucus vesiculosus* and *Ascophyllum nodosum*, with aqueous choline chloride:lactic acid systems reported as among the most effective for phlorotannin extraction [8]. Obluchinskaya et al. (2021) further applied ultrasound-assisted extraction with deep eutectic solvents (UAE-DES) to *F. vesiculosus* using lactic acid:choline chloride (3:1) and lactic acid:glucose:water (5:1:3), showing that DES composition and ultrasound time influence the recovery of antioxidant-rich extracts [83]. Obluchinskaya et al. (2023) optimized UAE-NADES extraction of Arctic *F. vesiculosus* using lactic acid:choline chloride (3:1), 30% water, 23 min extraction time, and a 1:12 sample-to-solvent ratio, with HPLC-HRMS/MS and MS/MS providing stronger molecular-level support than colorimetric screening alone [51].

**Figure 3:**
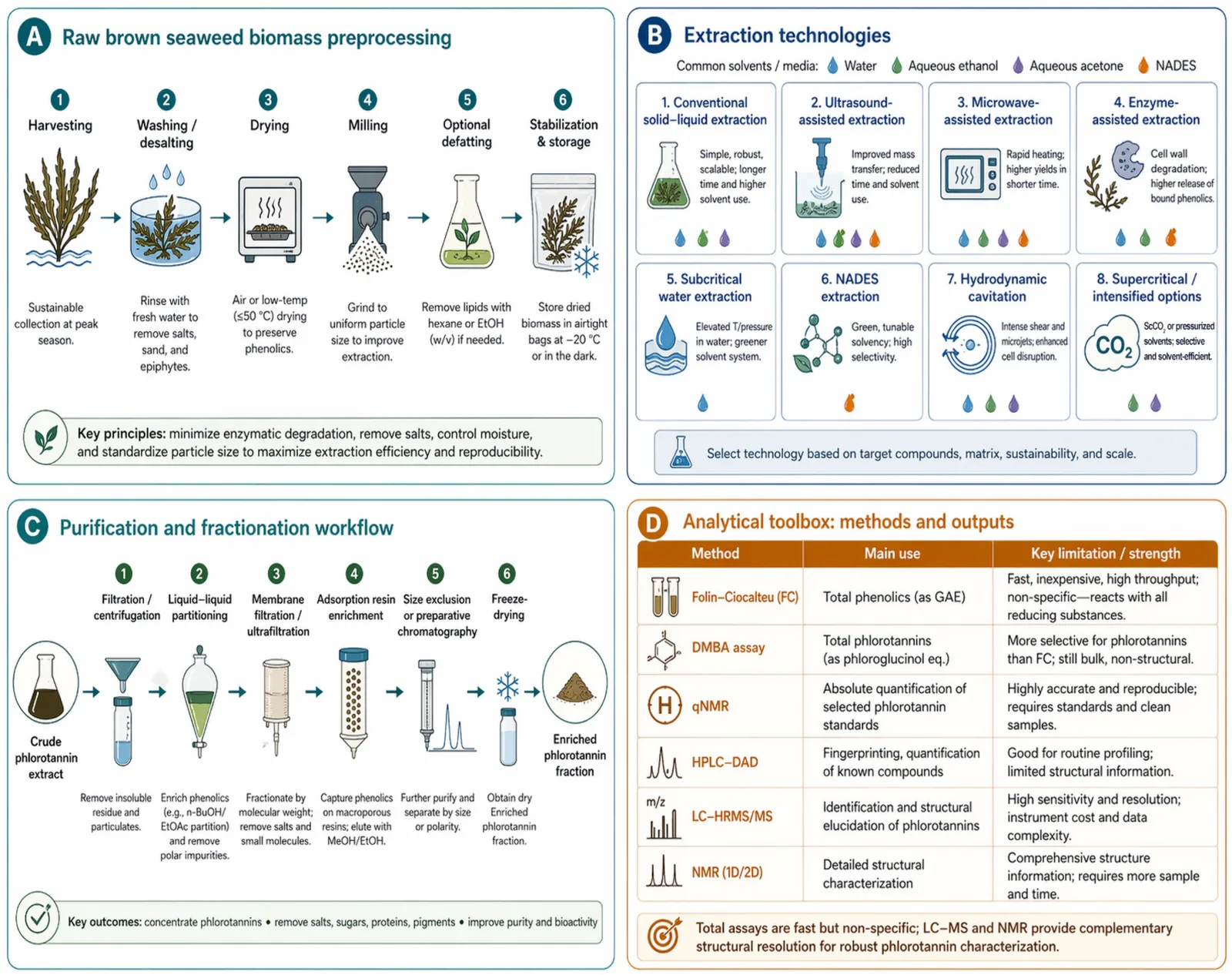
Integrated workflow for phlorotannin extraction, purification, and analytical validation from brown seaweeds. (**A**) Raw brown seaweed biomass preprocessing; (**B**) extraction technologies; (**C**) purification and fraction workflow; and (**D**) analytical toolbox: methods and outputs.

Table 2 extends the evidence to additional brown seaweed matrices, such as Mello et al. (2025), who used DES systems based on N,N-dimethylaminoethanol:benzyl alcohol and N,N-dimethylaminoethanol:1,3-propanediol for residual *Sargassum filipendula*, indicating that DES extraction may contribute to residual biomass valorization and bioactive compounds, including phlorotannins [57]. Praveen et al. (2026) reported NADES-UAE for *Ecklonia radiata*, in which choline chloride:lactic acid (1:3) with 20% water was the most effective solvent tested for phlorotannin extraction [102]. Zeb et al. (2024) applied water-rich-NADES-UAE to *Saccharina latissima* for polyphenol extraction using betaine: 1,3-butanediol (1:1), a 1:20 biomass-to-solvent ratio, and extraction for 1 h at 50 °C [50]. FC-derived values should be interpreted within the limitations discussed in Section 4.1. Advances in the literature not only concern the existence of NADES but also their coupling with process intensification, such as UAE paired with water-rich NADES, which is particularly significant because it addresses both solvent and mass-transfer limitations simultaneously [50]. The UAE can disrupt boundary layers, facilitate solvent penetration, and accelerate the release of phenolic material from the cell–matrix, while the WR-NADES medium improves solubility and protects the target compounds from aggressive oxidative conditions [50]. From a scale-up perspective, this addresses a common criticism of green solvents, namely their relatively high viscosity and slow mass transfer. NADES-UAE partly answers that critique by compressing extraction time and lowering the temperature burden [51,102]. The integration of NADES with UAE, MAE, PLE, SCCO2, EAE, and SLE extraction is particularly important for phlorotannin studies in brown seaweeds. Figure 3 summarizes this extraction logic as a workflow; the process begins with biomass handling, harvesting, washing, desalting, drying, milling, and storage, all of which affect phlorotannin stability before extraction. The next step is solvent-assisted release, which may involve ethanol–water, acetone–water, NADES with UAE, MAE, PLE, EAE, or combined systems. The extract then requires enrichment or purification. This step separates phlorotannins from matrix components, and finally, analytical validation confirms whether the recovered fraction is truly phlorotannin-rich.

**Table 2:** Extraction, cleanup, separation, detection, and quantification strategies for brown seaweed phlorotannins.

Ref./Study	Species/Location	Extraction Method	Clean-Up Method	Separation/Characterization	Detection Technique	Quantification/Bioactivity
[57]	Residual *Sargassum filipendula*	DES extraction using N,N-dimethylamino-ethanol:benzyl alcohol 1.30:1 and N,N-dimethylamino-ethanol:1,3-propanediol 1.83:1; 68.4–74.4 °C selective, 120 °C maximum pigment/phlorotannin extraction	np	Optimization of the DES process	FC assay	TPC: F–C, UV-Vis DPPH, ABTS, FRAP
[51]	*Fucus vesiculosus*	NADES-UAE of phlorotannins. (lactic acid:choline chloride; 3:1) provides a high yield of phlorotannins under the following extraction conditions: extraction time 23 min, 30.0% water concentration and 1:12 sample to solvent ratio.	np	HPLC-HRMS and MS/MS technique	MS/MS	TPC: F–C, UV-Vis
[102]	*Ecklonia radiata*	NaDES-UAE solvents tested, choline chloride:lactic acid (molar ratio of 1:3) supplemented with 20% water, was the most effective for the extraction of phlorotannin.	np	UPLC-MS/MS	MS/MS	TPC: F–C, UV-Vis
[50]	*Saccharina latissima*	UAE-WRNADES betaine and 1,3-butanediol (1:1) 1:20 *w*/*w* biomass to solvent ratio and a 1 h extraction time at 50 °C.	XAD-07 with ethanol and water washing	qNMR	NMR	TPC: F–C, UV-Vis
[83]	*Fucus vesiculosus*	Frozen samples thawed for 2 h at room temperature, and then mixed at a ratio of 1:10 (*w*/*v*) with NADES Lactic acid:Choline chloride 3:1 and Lactic acid:Glucose:H_2_O 5:1:3. The UAE was performed for 20 and 60 min.	np	Optimization of UAE-DES process for antioxidant activity	FC assay	TPC: F–C, UV-Vis
[8]	*Fucus vesiculosus* L. and *Ascophyllum nodosum* (L.)	The extraction efficiency of polyphenols was evaluated using 10 NADES based on choline chloride, lactic acid, betaine, and glucose in various mole ratios. The effect of H_2_O on the extraction of phlorotannins from aqueous solutions of NADES was studied.	np	Optimization of the NADES process for extraction of phlorotannin	Folin–Ciocalteu	TPC: F–C, UV-Vis
[153]	Fresh and storm-cast *Ascophyllum nodosum*, White Sea, Russia	Freeze-dried, powdered algae (0.5 grams of sample) were suspended in 5 mL of 70% acetone and subjected to continuous shaking in a 360-degree rotating shaker (Bio RS-24 BioSan, Latvia) for one hour at 4–6 °C.	np	FTIR + UV/Vis method validation	FC assay at 750 nm, 45 min, phloroglucinol standard	FTIR and TPC: F–C, UV-Vis
[139]	*Fucus vesiculosus*, Iceland	SLE, 80% aqueous ethanol, 200 rpm, 24 h	Extraction with ethyl acetate	UHPLC; 3 μm ODS-3, 150 × 2 mm column	DAD-ECD- QTOFMS	—
[129]	*Undaria pinnatifida*, China	PLE (52% ethanol in water; 5.2 h heating at 170 °C)	np	UPLC	Triple-TOF/MS (TOF MS/MS)	TPC: F–C, UV-Vis
[154]	*Undaria pinnatifida*, Japan	SLE (distilled water; 10 min constant shaking in water bath at 80 °C)	np	HPLC; column (C18 Kinetex; 150 mm × 4.6 mm × i.d. 2.6 μm) Mobile phase; water-acetic acid (99:1, *v*/*v*) water-acetonitrile-acetic acid (67:32:1, *v*/*v*/*v*)	DAD	TPC: F–C, UV-Vis
[116]	*Sargassum fusiforme*, China	SLE, 52% ethanol/water, 50 °C, 3 h	Ethyl acetate extraction	UHPLC, Poroshell 120 EC-C18	DAD-ESI-QTOF-MS	TPC: F–C, UV-Vis
[110]	*Sargassum muticum* (1) Norway (2) Portugal (3) Ireland (4) Spain (5) France	PLE (ethanol: water (95:5); 10.3 MPa for 20 min at 160 °C)	Multi-step purification; (1) PLE samples were re-dissolved in water then LL extraction with dichloromethane (2) acetone and ethanol extraction	LC × LC D1; Lichrospher diol-5 column (150 × 1.0 mm, 5 μm) D2; C18 (50 × 4.6 mm, 2.7 μm) Mobile phase	D1; DAD D2; Ion trap MS	TPC: F–C, DMBA (UV–Vis)
[141]	*Laminaria digitata*, Scotland	SLE (80% aqueous methanol; macroalgae were bead-milled for 1.5 h, then filtered over a cellulose filter)	Extraction with ethyl acetate and normal phase-flash chromatography using hexane, acetone and methanol as eluents	Reversed phase-UHPLC; C18 column (2.1 mm × 150 mm i.d., particle size 1.7 μm) Mobile phase; Acetonitrile and water containing 0.1% *v*/*v* formic acid	DAD-ESI-MS, MALDI TOF MS and NMR	TPC: DMBA and F–C (UV–Vis), NMR
[155]	*Sargassum muticum*, France	(1) EAE (0.1 M sodium acetate- acetic acid buffer; viscozyme; 2 h at 50 °C/pH 4.5). (2) PLE (ethanol: water (25:75, *v*/*v*) for 20 min at 120 °C). (3) EAE-PLE (0.1 M phosphate buffer; alcalase; 2 h at 50 °C/pH 7.0 +ethanol: water (25:75, *v*/*v*) at for 20 min at 120 °C) (4) EAE-PLE (0.1 M sodium acetate-acetic acid buffer; viscozyme; 2 h at 50 °C/pH 4.5+ethanol: water (25:75, *v*/*v*) for 20 min at 120 °C)	np	HPLC; XDB-C18 column (4.6 × 150 mm, 5 μm) Mobile phase; Water (0.1% formic acid) and acetonitrile	DAD-Ion trap MS	TPC: DMBA, F–C (UV–Vis)
[76]	*Ascophyllum nodosum*, Iceland	SLE (aqueous ethanol 30% (i); at 25 °C in 30 min, aqueous ethanol 80% (ii); 20 h at 25 °C +10 h at 65 °C)	Two-step purification by two SPE cartridges using 100% acetonitrile and 0.25% aqueous acetic acid	UHPLC; CSH Phenyl-hexyl UPLC column (2.1 mm × 100 mm, 1.7 μm, 130 Å) Mobile phase; water and acetonitrile/water (95/5, *v*/*v*) both containing 10 mM ammonium formate	DAD-ESI- Ion-trap MS	TPC: F–C, UV-Vis
[138]	*Ascophyllum nodosum*, Ireland	SLE, cold water; ethanol/water, shaking, room temperature	SPE and HPLC-grade water fractionation	UPLC-PFP and UPLC-C18	TQD-MS	—
[68]	*Carpophyllum flexuosum*, New Zealand	(1) MAE (Milli-Q water; the dried and milled sample extracted in a focused microwave reactor at 160 °C for 3 min) (2) SLE (sequential solvent mixtures; methanol: water (1:1), acetone: water (7:3), both mixtures at pH 2 controlled by HCl	Dichloromethane partition, ethyl acetate extraction	HPLC-C18, 250 × 4.6 mm, 5 µm	DAD-ESI-MS, NMR	TPC: F–C, UV-Vis
[147]	*Fucus vesiculosus* Ireland	SLE (ethanol: water (80:20); shaking at 150 rmp and room temperature for 24 h)	Multi-step purification: (1) partitioned with HPLC-grade water (2) fractionated by Molecular Weight Cut-off Dialysis (3) fractionated using a two-step Reversed- Phase Flash Chromatography	UPLC-PFP, water/acetonitrile/formic acid	TQD-MS	TPC: F–C, UV-Vis
[107]	*Saccharina latissima*	PLE and MAE with GRAS solvents; MAE: 80 °C, 2% EtOH/water, 40 mL/g; PLE2: 80 °C water	np	Optimization of PLE and MAE for phlorotannin	FC assay	TPC: F–C, UV-Vis
[62]	*Durvillaea incurvata*	USAE with 32.5% ethanol–water	Diaion HP-20 resin purification	FTIR, HPLC-IR, UHPLC-QToF-MS/MS	Spectrophotometry and MS/MS	DMBA colorimetric method
[156]	*Dictyopteris justii*	UAE optimized by Box–Behnken: 70% ethanol, 1:10 solid/liquid, 25 °C, 20 min	np	Chromatographic indication of hydroxypentafuhalol-B	DMBA colorimetric method	DMBA colorimetric method

## 4. Selective Quantification and Analytical Validation of Phlorotannins

### 4.1. Limitations of Folin–Ciocalteu and Bulk Colorimetric Assays

The FC assay remains useful for rapid screening but should not be interpreted as a phlorotannin-specific method because it measures total reducing capacity and is susceptible to interference from non-phenolic reducing compounds. Throughout this review, FC-derived values are therefore considered screening indicators rather than direct measures of phlorotannin concentration [13,56,66,102,141,142]. In crude brown seaweed extracts, co-extracted sugars, proteins, pigments, salts, ascorbic acid, amino acids, and other reducing compounds may bias TPC values, especially when comparing different species, extraction solvents, or purification levels [8,14,51,59,153]. qNMR shifts the quantitative discussion from color response to proton-counting physics [58].

### 4.2. Why qNMR Matters for Phlorotannin Quantification

**Figure 4:**
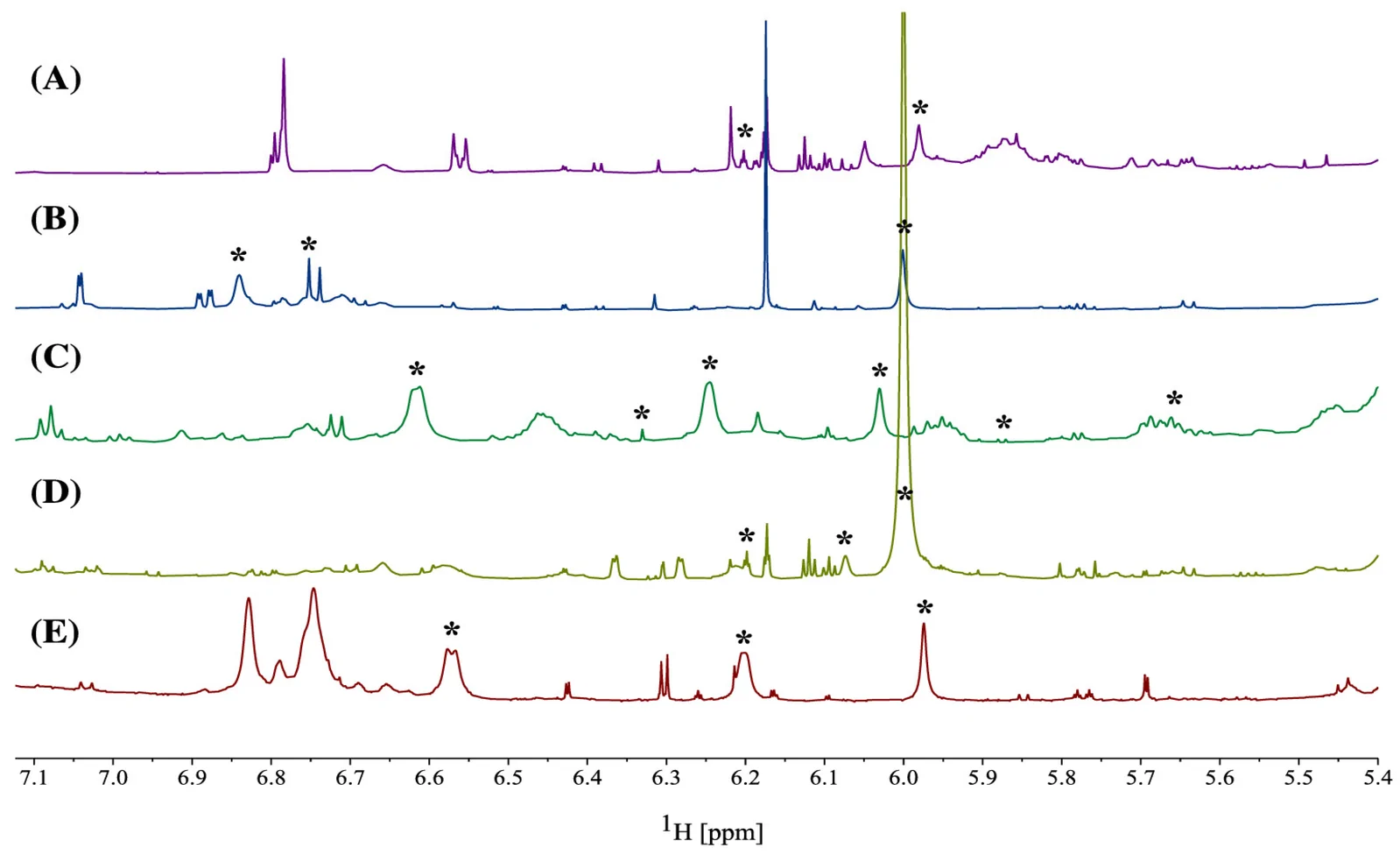
Selective qNMR quantification of brown seaweed polyphenols compared with total polyphenol content assays. Advancing quantification methods for polyphenols in brown seaweeds—applying a selective qNMR method compared with the TPC assay. ^1^H NMR spectra displaying the polyphenolic region (7.0–5.5 ppm) used for quantification of *F. vesiculosus* (**A**), *A. nodosum* (**B**), *S. latissima* (**C**), *L. digitata* (**D**), and *L. hyperborea* M20 (**E**). Signals labelled with asterisk (*) were deselected based on 2D NMR prior to quantification [58].

Selective qNMR can provide a chemically traceable approach for phlorotannin quantification because selected and assigned aromatic resonances are integrated against an internal or external reference standard, rather than relying on colorimetric response, molar absorptivity, or external calibration curves used in conventional assays (Figure 4) [58,59,157,158]. However, this approach should not be considered assumption-free or automatically absolute. Reliable quantification requires sufficient relaxation delay, good baseline quality, signal resolution, correct integration, sample purity, solubility, and accurate resonance assignment [58]. As shown in the reported polyphenol quantification case study of *Ulva intestinalis*, the NMR integral is converted into concentration by dividing the selected signal area by the assumed number of aromatic protons contributing to that signal [59]. Therefore, if the number of aromatic protons is misassigned, the calculated molar concentration, mass concentration, and final yield may be directly overestimated or underestimated. For phlorotannins, different oligomers and linkage types may contain different numbers of observable aromatic protons, making accurate proton assignment essential [59]. For structurally heterogeneous phlorotannins, additional uncertainty arises from overlapping aromatic signals, variation in oligomer size, assumptions about average molecular weight, and the estimated number of aromatic protons contributing to the selected integration region [58,59,157,158,159].

Although qNMR is less sensitive than UV–Vis/colorimetric assays, it is most reliable for extracts or purified fractions containing sufficient phlorotannin levels, rather than for trace-level detection. For example, Jégou et al. (2015) demonstrated the use of NMR for targeted quantification of phloroglucinol, the main phenolic/phlorotannin-related compound in *Cystoseira tamariscifolia* [157]. The method used TSP as an internal standard and quantified the phloroglucinol singlet at 6.02 ppm in D_2_O, with a long relaxation delay to support quantitative integration. The study reported at least 94.2% accuracy for standard phloroglucinol solutions and showed that FC generally yielded higher values than qNMR, mainly because FC can detect non-phenolic reducing compounds [157]. Although qNMR has already been applied for selective quantification of brown seaweed polyphenols, its future development for phlorotannin-specific quantification should move beyond bulk integration of the aromatic region. A promising strategy is to use 2D NMR, particularly HSQC/HMBC, as a prior structural-filtering step to define which resonances belong to phlorotannin subclasses before quantitative integration [58,59,66,157]. HMBC-assisted assignment can distinguish aryl–aryl, diaryl-ether, oxygenated phenolic, and dibenzodioxin-type regions, thereby helping to select signals associated with fucols, phlorethols/fucophlorethols, fuhalols, and eckol/carmalol-type compounds [66].

### 4.3. qNMR Versus Folin–Ciocalteu, and LC-HRMS/MS

Each analytical method answers a different question; for example, FC asks how reducing capacity behaves like phenolics under the assay conditions, while LC-HRMS/MS identifies masses and fragmentation patterns, often with high sensitivity but limited certainty for high-order isomers. qNMR quantifies the signal from a chemically defined region in molar terms (Table 3) [50,56,58,59,142]. The comparison shows that FC and qNMR agree reasonably for cleaner or lower-phenolic Laminariaceae samples, but FC may overestimate phenolic content in Fucaceae. For *L. hyperborea* and *L. digitata*, qNMR gave 0.4–0.6% DW, while TPC gave 0.6–0.8% PGE/DW; for *S. latissima*, qNMR gave 1.2% and TPC 1.5%. In contrast, *F. vesiculosus* showed 1.1% by qNMR but 4.1% by TPC, and *A. nodosum* showed 0.9% by qNMR but 2.0% by TPC, indicating up to three-fold overestimation by FC in eulittoral Fucaceae [58]. The *Ulva intestinalis* case study further demonstrates that total phenolic quantification depends strongly on method assumptions: qNMR gave 5.5% DW, HPLC-DAD 1.1% DW, and TPC only 0.4% DW, despite using gallic acid as reference in all methods [59].

The observed differences among qNMR, FC, and HPLC-DAD measurements highlight that these methods provide complementary information and should not be considered interchangeable analytical tools. For *L. hyperborea* side-streams, 60% aqueous methanol/ethanol maceration produced comparable phenolic recovery to UAE and ASE, while purification by PuriFlash and semi-preparative HPLC increased phenolic selectivity, qNMR/TPC values, and ORAC antioxidant activity. Importantly, the study identified low-molecular-weight phenolic acids, phlorotannin oligomers, sulfated phenolics, and non-phenolic co-extractives, reinforcing the need to combine qNMR with LC-MS/MS and purification before making phlorotannin-specific claims [142]. FC-derived values should be viewed as screening estimates rather than selective phlorotannin quantification [58].

## 5. Biological Activities and Translational Roadmap for Ageing-Related Phlorotannin Applications

Brown seaweed phlorotannins exhibit a broad spectrum of biological activities, including antioxidant, anti-inflammatory, antimicrobial, antiviral, antidiabetic, anti-adipogenic, anticancer, neuroprotective, cardiometabolic, and skin-protective effects, but their translational value depends on matching each activity with the appropriate evidence level, extract standardization, bioavailability profile, and application route [3,13,16,24,28,29,37,41,49,82,92,95,98,99,109,110,147,160,161]. For ageing-related research, these activities are best organized around convergent mechanisms rather than isolated disease labels, because oxidative stress, chronic low-grade inflammation, metabolic dysregulation, advanced glycation, endothelial dysfunction, mitochondrial vulnerability, extracellular-matrix degradation, and impaired cellular resilience are interconnected drivers of biological ageing [13,26,29,30,33,38,129,156]. Unless specifically noted, most reported biological effects are supported primarily by in vitro studies and preclinical animal models. This limitation applies broadly across the current phlorotannin literature [22,29,31,38,39,40,87,154].

### 5.1. Bioavailability and Gastrointestinal Fate

From a translational perspective, phlorotannins are attractive but technically demanding marine bioactives because their stability, gastrointestinal fate, solubility, oral bioavailability, sensory compatibility, formulation behavior, and regulatory acceptability remain incompletely resolved [3]. Following ingestion, phlorotannin stability and bioaccessibility are influenced by gastrointestinal pH, digestive enzymes, bile salts, molecular size, and interactions with the food matrix, with simulated digestion studies showing substantial compositional changes while retaining some antioxidant and anti-inflammatory activity [86,162]. Larger, poorly absorbed phlorotannins may reach the colon for microbial transformation into smaller metabolites, followed by glucuronidation or sulfation after absorption; however, current evidence remains insufficient to define absolute bioavailability and complete pharmacokinetic parameters [3,13,14,86,162]. Consequently, the literature contains many promising in vitro datasets but comparatively few standardized in vivo or human studies using chemically characterized preparations [3,29,37,38]. Future progress will therefore require integration of ecological standardization, green extraction, selective quantification, purification logic, mechanistic bioassays, pharmacokinetic evaluation, delivery-system design, and scalable marine biorefinery strategies [3,41,56,58]. However, these applications should be viewed as longer-term pharmacological opportunities rather than near-term functional-food claims because convincing evidence of absorption, metabolism, blood–brain barrier permeability, target engagement, and clinical efficacy remains limited [4,14,66,93,99]. Although phlorotannins show promising antioxidant, anti-inflammatory, anti-glycation, enzyme-modulatory, neuroprotective, and skin-ageing activities, most claims remain preclinical and require stronger evidence on bioavailability, metabolism, dose standardization, formulation stability, and clinical relevance. Near-term translation is most realistic for topical cosmeceuticals and gut-localized metabolic applications, whereas systemic anti-ageing and neurocognitive uses require more extensive pharmacokinetic and clinical validation.

### 5.2. Antioxidant and Redox-Buffering Functions

Antioxidant activity is the most widely reported bioactivity of phlorotannins, reflecting their hydroxyl-rich structures and capacity for radical scavenging, reducing power, and redox buffering [11,89,129,143,147]. However, antioxidant performance is not determined solely by hydroxyl number or polymer size; oligomerization state, linkage type, substitution pattern, molecular conformation, fraction purity, and assay system also strongly influence measured activity [13,163]. Oxidative stress contributes to inflammation, glycation, mitochondrial dysfunction, and tissue degeneration. Antioxidant activity alone is therefore insufficient to support translational claims [13,30,99]. Kim et al. (2009) isolated phlorofucofuroeckol A, dieckol, and dioxinodehydroeckol from the brown seaweed *E. stolonifera* using bioactivity-guided fractionation supported by NMR and mass spectrometry [20]. These compounds showed strong radical-scavenging activity, and phlorofucofuroeckol A and dieckol significantly reduced intracellular reactive oxygen species in LPS-stimulated RAW 264.7 macrophages [20]. Thus, *E. stolonifera* phlorotannins provide mechanistic evidence for modulation of oxidative-stress-linked inflammatory responses relevant to ageing biology.

### 5.3. Antidiabetic and Metabolic-Ageing Applications

Metabolic ageing is a plausible application area because several mechanisms relevant to glucose homeostasis can be investigated using digestive enzyme, oxidative stress, and metabolic models. Current evidence suggests that phlorotannins may inhibit carbohydrate-hydrolysing enzymes such as α-amylase and α-glucosidase, attenuate postprandial glycaemic responses, reduce oxidative damage, and modulate pathways associated with insulin sensitivity and metabolic stress [13,27,36,61]. For example, Gheda et al. (2021) evaluated phlorotannins extracted from the brown seaweed *Cystoseira compressa* in streptozotocin-induced diabetic rats [36]. The extract was characterized using the DMBA assay, UV spectroscopy, FTIR, UPLC-MS/MS, and GC-MS, and the detected phlorotannin constituents were mainly assigned to the fuhalol class. In diabetic rats, treatment with the extract reduced serum glucose, hepatic α-amylase and α-glucosidase activities, and liver malondialdehyde, while improving serum insulin, hepatic glutathione, total antioxidant capacity, and skeletal-muscle AMPKα2 expression. Histological analysis also indicated reduced pancreatic β-cell damage compared with untreated diabetic animals [36]. These studies indicate that phlorotannins may influence diabetes-related metabolic dysfunction through enzyme inhibition, antioxidant effects, and AMPK-associated mechanisms [27,36].

### 5.4. Anti-Inflammatory and Inflammaging Relevance

Inflammaging represents an important translational axis because chronic low-grade inflammation contributes to metabolic decline, vascular dysfunction, neurodegeneration, and skin ageing [20,23,37,143,162]. Mechanistic studies suggest that phlorotannins can modulate inflammatory networks involving NF-κB, MAPK, JAK/STAT3, and NLRP3-associated signalling, thereby reducing cytokine production, oxidative amplification, and tissue-injury responses [23,129,143,162]. Catarino et al. (2022) investigated phlorotannins from the brown seaweed *H. elongata*, which contained fucophlorethol- and carmalol-type compounds, and evaluated how simulated gastrointestinal digestion affected their antioxidant and anti-inflammatory properties [162]. Although digestion reduced measurable antioxidant capacity and altered the phlorotannin profile, the digested extract still showed strong inhibition of nitric oxide production in LPS-stimulated RAW 264.7 macrophages. These findings suggest that *H. elongata* phlorotannins, or their digestion-derived products, may retain intracellular anti-inflammatory activity even after gastrointestinal transformation. Together with studies on *A. nodosum* and *F. vesiculosus* aqueous extracts, which reduced LPS-induced nitric oxide production, cytokine/chemokine release, TLR4-dependent NF-κB activation, and intestinal barrier dysfunction, these findings support the anti-inflammatory relevance of brown seaweed phenolics in ageing-related biology [20,23,41,84,129,143].

### 5.5. Anti-Glycation, Protein Carbonyl Stress, and Ageing

Anti-glycation activity should be considered a central ageing-related mechanism rather than a secondary antioxidant effect, because advanced glycation end products (AGEs) contribute to diabetes, vascular stiffening, renal injury, skin ageing, and neurodegenerative processes [4,7,13,14,30,66]. Phlorotannins are relevant in this context because they may inhibit AGE formation, scavenge reactive carbonyl intermediates, and attenuate oxidative-inflammatory amplification associated with glycation damage [4,14,23,30,33,129,162]. Together, these effects link metabolic ageing, vascular ageing, renal dysfunction, skin ageing, and neurodegeneration, but it requires better fraction-level characterization and more physiologically relevant models [6,7,17,28,31,129]. The study by Liu and Gu (2012) showed that phlorotannin-rich extracts from *F. vesiculosus* inhibited AGE formation in glucose- and methylglyoxal-induced bovine serum albumin models [30]. The authors extracted phlorotannins with 70% acetone and fractionated the extract into dichloromethane, ethyl acetate, butanol, and aqueous fractions; the ethyl acetate fraction showed stronger anti-glycation activity than the crude extract [30]. Mechanistically, this activity was attributed partly to scavenging of reactive carbonyl species, particularly glyoxal and methylglyoxal. These findings support *F. vesiculosus* phlorotannins as promising marine-derived anti-glycation candidates for ageing-related functional food, nutraceutical, and cosmeceutical applications, although better fraction-level characterization, physiologically relevant models, and in vivo or clinical validation remain necessary.

### 5.6. Neurocognitive Ageing and Skin-Ageing Products as Differentiated Development Tracks

Neurocognitive ageing is a promising but higher-risk translational track for phlorotannins, and the strongest mechanistic evidence currently comes from *Ecklonia*-derived compounds tested in antioxidant, anti-neuroinflammatory, anti-apoptotic, cholinergic, and amyloid-related models [20,22,41,47,92,93,95,97,99,100,102,105,164]. Lee and Jun (2019) evaluated eckol, dieckol, and 8,8′-bieckol from the brown seaweed *Ecklonia cava* against Alzheimer’s disease-related targets [96]. These phlorotannins inhibited both β-secretase 1 (BACE1), which is involved in amyloid-β production, and acetylcholinesterase (AChE), which is associated with cholinergic dysfunction. Among the tested compounds, 8,8′-bieckol showed the strongest dual inhibitory activity against BACE1 and AChE, supported by in vitro assays and in silico docking [96]. These issues are discussed in the bioavailability section.

Skin ageing and cosmeceutical development represent a comparatively realistic translational route because topical delivery can partly bypass oral bioavailability constraints [28,33,35,91,121,165,166,167]. Current cosmeceutical studies support the multifunctional potential of brown seaweed phlorotannins, particularly as antioxidant, anti-inflammatory, skin-brightening, anti-wrinkling, and extracellular-matrix-protective agents [7,28,113,166,167]. A representative example is the study by Sharma and John (2023), who evaluated *Padina tetrastromatica* as a brown macroalgal source of photoprotective and anti-ageing compounds [167]. The ethyl acetate fraction was more enriched in phlorotannins than the aqueous fraction and contained phloroglucinol-derived oligomers, including diphlorethol/difucol, fucophlorethol, fucodiphlorethol, fucotriphlorethol, and fucophlorethol hexamer. Functionally, this fraction showed antioxidant activity, UVA absorbance, a high SPF value, no cytotoxicity toward SK-MEL-28 skin cells up to 100 μg/mL, and inhibition of tyrosinase, elastase, and collagenase [167]. These findings support *P. tetrastromatica* phlorotannins as promising marine-derived cosmeceutical candidates, but their translational value should not be inferred solely from crude antioxidant activity.

**Figure 5:**
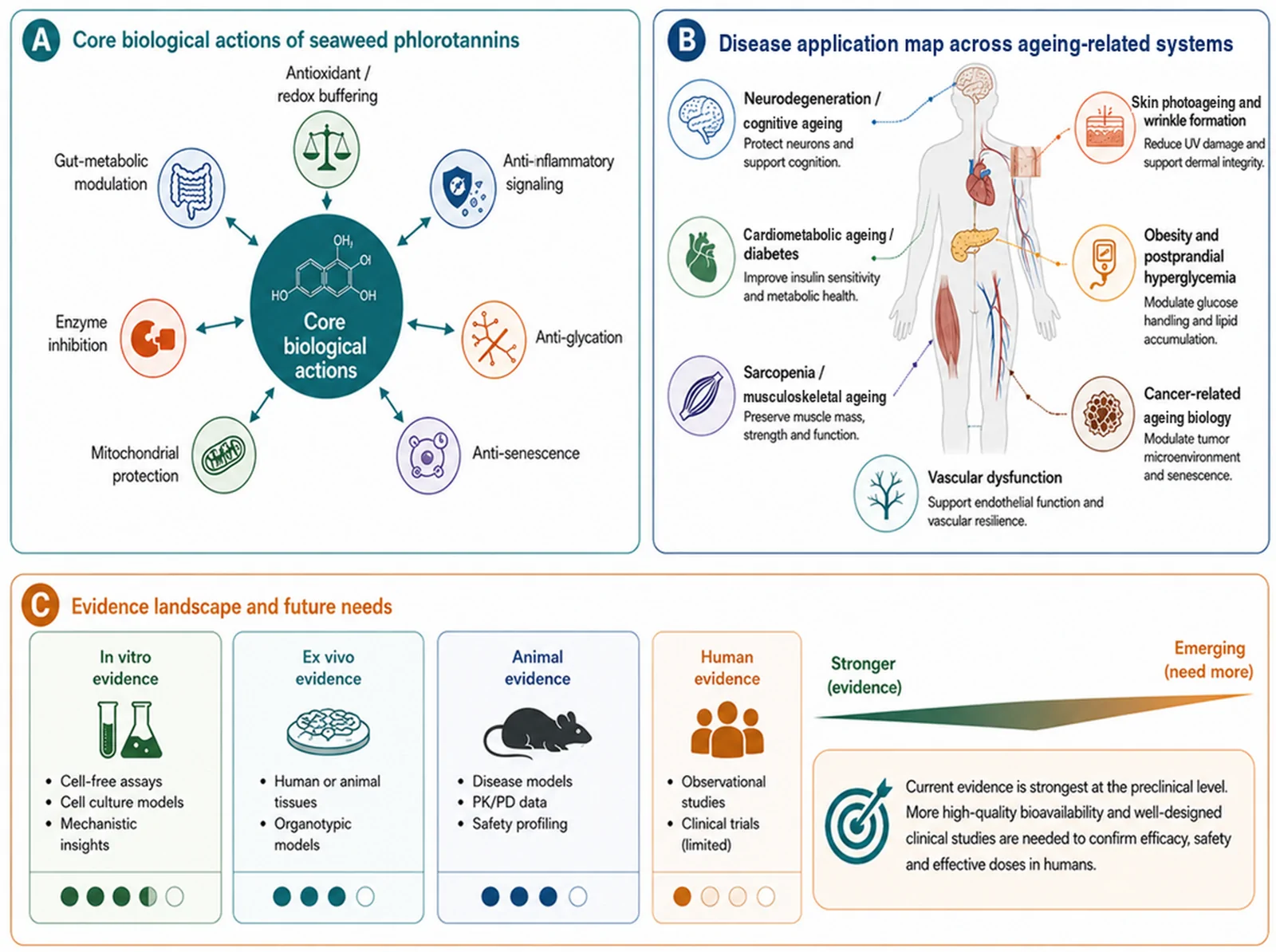
Ageing-related biological targets and translational evidence landscape for seaweed phlorotannins [4,9,22,28,33,36,82,101,162,169]. (**A**) Core biological actions of phlorotannins; (**B**) disease application map across ageing-related systems; and (**C**) evidence landscape and future needs.

Further development requires chemically standardized extracts, mechanism-aware skin models, formulation stability testing, residual-solvent and safety assessment, sensory acceptability, and in vivo or clinical validation [4,7,26,28,87,161,167]. Table 4 summarizes the biological activities of phlorotannins across different brown seaweed species. It shows that ageing-related effects are not restricted to a single species or mechanism. For example, *F. vesiculosus* is linked with prebiotic effects and gastrointestinal health, while *C. compressa* has been reported to exhibit antidiabetic activity, and *E. cava* is strongly represented in anti-melanogenesis and skin-related studies. Figure 5, which organizes phlorotannin bioactivity around aging-related disease systems and mechanisms, connects antioxidant, anti-inflammatory, anti-glycation, anti-diabetic, neuroprotective, skin-protective, and cardiometabolic effects with common aging pathways.

Although phlorotannins show antioxidant, anti-inflammatory, antidiabetic, anti-glycation, neuroprotective, photoprotective, and skin-protective activities, the translational evidence remains uneven [1,2,4,5,7,66]. Most mechanistic evidence is still derived from chemical assays, enzyme-inhibition tests, or cell-based models. Animal studies provide important preclinical support for metabolic, inflammatory, neuroprotective, hepatoprotective, and skin-related effects, but many studies use crude or partially enriched extracts rather than chemically defined phlorotannin fractions [2,4,11]. Interpretation should consider extract composition and purification level.

## 6. Phlorotannin-First Brown Seaweed Biorefinery Concept

Industrial brown-seaweed valorization is currently led by polysaccharide-rich and carbohydrate-based product streams, particularly alginate, fucoidan, laminarin, mannitol, minerals, feed ingredients, fertilizers, and residual biomass products [106,127,144,175,176,177]. This is commercially logical because these fractions dominate biomass mass flow and already have clearer industrial relevance. In contrast, phlorotannins are usually present at lower biomass levels, but they may represent a higher-value functional stream because of their antioxidant, anti-inflammatory, anti-glycation, enzyme-inhibitory, and cosmeceutical potential [1,2,5,6,28]. Phlorotannins represent a complementary high-value phenolic stream that may strengthen the economic logic of brown-seaweed biorefineries when recovered early and selectively [5,11,13,127]. Therefore, a phlorotannin-first brown seaweed biorefinery should be presented not as an established industrial model but as a conceptual framework requiring experimental and techno-economic validation. Several industrial examples illustrate the transition from single-product seaweed processing toward integrated biomass utilization. Alginor ASA develops *Laminaria hyperborea* biorefining for kelp-based pharmaceutical and nutraceutical ingredients and describes upstream separation of kelp into leaf and stipe fractions (Alginor ASA. Upstream https://alginor.no/solutions/upstream/, accessed on 6 July 2026). Algea AS processes *Ascophyllum nodosum* into extracts and phytocomplexes for agriculture and animal feed (Algea AS. https://www.algea.com/index.php/52-links/227-fao?utm, accessed on 6 July 2026). AlgiPharma AS develops alginate oligomer technologies for biomedical applications, including patent-protected uses against biofilms and related indications (AlgiPharma AS. https://algipharma.com/publication/patents/?utm, accessed on 6 July 2026). These examples show that substantial process innovation exists beyond academic publications and that phlorotannin recovery should be considered within broader brown seaweed polysaccharide and biorefinery value chains.

**Figure 6:**
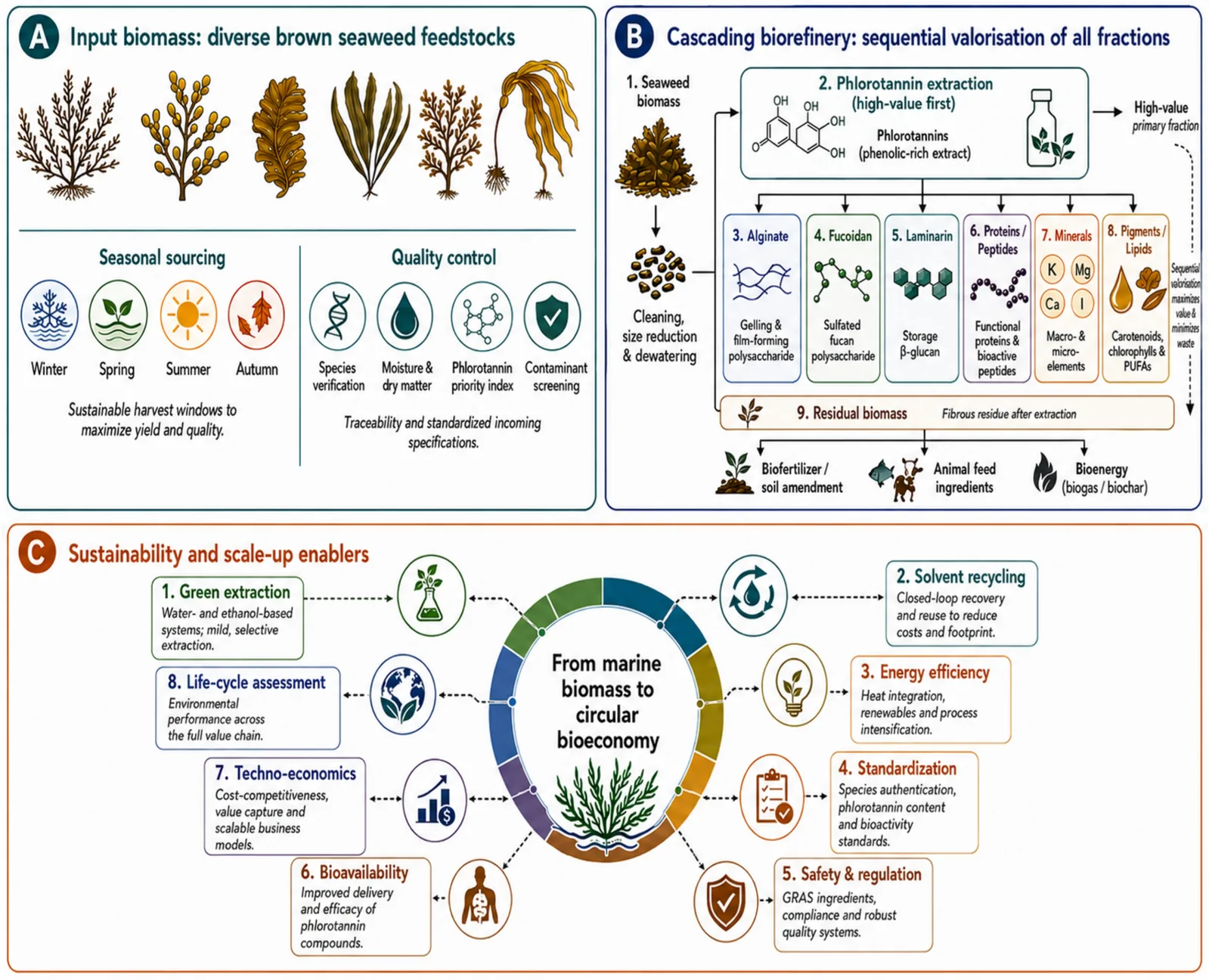
Phlorotannin-first brown seaweed biorefinery: high-value phenolic recovery and cascading biomass valorization [13,14,173,179]. (**A**) Input biomass: diverse brown seaweed feedstocks; (**B**) Cascading biorefinery: sequential valorisation of all fractions; and (**C**) sustainability and scale-up enablers.

Early phlorotannin recovery is justified by the susceptibility of phenolic structures to harsh alkaline, acidic, thermal, or prolonged enzymatic processing commonly used for downstream polysaccharide recovery. A realistic process would begin with carefully controlled biomass handling, including species selection, harvest documentation, washing, desalting, drying or fresh processing, size reduction, moisture control, and storage [13,49,145,178]. Mild extraction using food-compatible ethanol–water systems, water-rich NADES, ultrasound-assisted extraction, pressurized water/ethanol extraction, or other green technologies could then be applied before harsher alginate or mineral-processing steps [13,14,49,56,66]. However, extraction should not be judged only by total phenolic yield. Selectivity, structural preservation, solvent safety, solvent recovery, scalability, analytical reliability, and compatibility with downstream polysaccharide processing are equally important. The extraction, analytical characterization, and translational aspects of phlorotannins have been discussed in Section 3, Section 4 and Section 5. The residual biomass should then be sequentially valorized for fucoidan, alginate, laminarin, proteins, peptides, pigments, minerals, and final residual applications such as biofertilizers, soil amendments, fermentation substrates, or bioenergy [106,177]. Such a cascading strategy aligns with circular bioeconomy principles, but its feasibility cannot be assumed [106,177]. The major limitation is that early phlorotannin extraction adds processing steps, solvent-handling requirements, cleanup costs, and possible losses in downstream polysaccharide yield or quality. Industrial assessment requires quantitative data on phenolic yield per tonne of biomass, solvent consumption, solvent recovery, energy demand, product purity, and market value. Techno-economic analysis, life-cycle assessment, mass balance, solvent recycling, and pilot-scale validation are required before the phlorotannin-first model can be considered an industrially credible brown seaweed biorefinery strategy. Figure 6 proposes a process framework and identifies where phlorotannins could be protected and recovered early, but its feasibility depends on mass balance, solvent recovery, downstream polysaccharide quality, techno-economic performance, life-cycle impact, and product-specific regulatory requirements.

The economic viability of phlorotannin extraction depends on biomass cost, seasonal availability, drying and milling energy, solvent price, solvent recovery, extraction yield, purification intensity, analytical costs, product purity requirements, and final market positioning [127,179,180]. Conventional aqueous ethanol extraction remains attractive for food, nutraceutical, and cosmetic applications because ethanol is scalable, recoverable, and more acceptable than methanol or acetone [48,49,56,144]. Green adaptations such as ultrasound-assisted extraction, enzyme-assisted extraction, pressurized-liquid extraction, and NADES/DES-based extraction may reduce time, improve mass transfer, or lower solvent burden, but their industrial benefit depends on solvent recyclability, viscosity, downstream removal, regulatory acceptance, and compatibility with existing seaweed-processing infrastructure [11,48,55,56]. A circular-economy approach is therefore essential. In a phlorotannin-first biorefinery, the phenolic fraction is recovered early as a high-value stream, while the remaining biomass is sequentially processed for alginate, fucoidan, laminarin, mannitol, proteins, pigments, minerals, feed, fertilizers, soil amendments, fermentation substrates, biomaterials, or bioenergy (Alginor AS) [177,179]. This cascading strategy reduces waste, distributes processing costs across multiple product streams, and aligns phlorotannin recovery with the brown-seaweed polysaccharide industry.

## 7. Future Perspectives

Progress in brown seaweed phlorotannin research will require a shift from descriptive extract screening toward standardized, mechanism-oriented, and industrially realistic studies. The priority is species- and biomass-level standardization, as phlorotannin profiles are strongly influenced by taxonomy, season, tissue type, geography, environmental exposure, and post-harvest handling. The second priority is using advanced analytical methods that are increasingly improving phlorotannin characterization and quantification, as discussed in Section 4. The third priority is green extraction design, and WR-NADES and integration of NADES with UAE, MAE, EAE, and PLE should be compared using identical biomass lots and validated analytical endpoints, and the studies must mention the molar ratio, water content, viscosity, pH, extraction time, temperature, biomass-to-solvent ratio, solvent recovery, and potential assay interference.

The fourth priority is bioactivity validation, while many reported bioactivities are promising but remain dominated by in vitro models and poorly standardized extracts. Translational research should focus on chemically characterized preparations and clinically relevant validation strategies. The fifth priority is the biorefinery integration of phlorotannins, as they should be studied not only as isolated, extractable compounds but also as a high-value stream within brown seaweed cascading valorization. The studies should link phlorotannin extraction to the recovery of alginate, fucoidan, laminarin, proteins, pigments, minerals, and residual biomass. TEA, LCA, solvent recycling, regulatory acceptability, and product formulation should become routine components of applied phlorotannin research. A key limitation of the field is poor comparability among studies due to differences in species, harvest season, biomass pretreatment, extraction conditions, purification depth, and analytical method. A minimum reporting checklist should therefore include species, origin, season, drying method, solvent composition, extraction parameters, cleanup strategy, quantification method, and bioassay dose basis.

## 8. Conclusions

Brown seaweed phlorotannins are chemically distinctive marine polyphenols with substantial biological and industrial potential. Their translation, however, requires a shift from crude-extract descriptions toward selective extraction, cleaner fractionation, orthogonal structural characterization, and robust quantification. Emerging technologies, including advancements in NADES with UAE, MAE, PLE, and EAE-based extraction, offer greener and more targeted recovery but should be evaluated on selectivity, structural preservation, solvent safety, scalability, and analytical reliability rather than yield alone. Bioavailability remains a major translational bottleneck. A phlorotannin-first biorefinery strategy could preserve these high-value phenolics before harsher downstream biomass processing, enabling sequential valorization of polysaccharides, proteins, pigments, minerals, and residual biomass. Future progress depends on chemically standardized and analytically validated phlorotannin systems.

## Figures and Tables

**Table 3:** Comparative strengths and limitations of qNMR and TPC assays for phlorotannin quantification.

Evaluation Criterion	qNMR Approach	TPC Assay
Sample preparation	Dry-weight-based dilution, commonly in DMSO; the sample is recovered after analysis	Dry-weight-based dilution, commonly in MeOH; no sample recovery after reaction
Detection principle	Direct structural measurement of proton resonances	Colorimetric, reaction-dependent measurement
Calibrant/reference	Certified reference material, such as TMS; internal or external standard	Structurally identical or equivalent phenolic reference compound
Quantification basis	Signal integration relative to the standard	Calibration curve generated from the reference standard
Main assumption	The number of protons per aromatic ring in the sample is known or estimated.	Polyphenol concentration is proportional to color development.
Selectivity/specificity	More structurally informative; affected by resonance overlap, improved by ^13^C and 2D NMR optimization	“All-in-one” assay; non-selective toward phlorotannins because other reducing compounds may contribute
Reproducibility	Instrument-dependent but less affected by chemical reaction variability	Work-up- and reaction-dependent
Main strength	Direct, structure-oriented quantification	Simple, rapid, inexpensive screening method
Main limitation	Requires NMR instrumentation and careful spectral interpretation	Indirect and non-specific for phlorotannins

**Table 4:** Reported bioactivities, mechanisms of action, and key findings for brown seaweed phlorotannins.

Name of Brown Seaweed	Activities	References
*Fucus vesiculosus*	A prebiotic effect contributes to the maintenance of healthy gastrointestinal conditions.	[86]
*Cystoseira compressa*	Anti-diabetic activity	[36]
*Ecklonia cava*	Anti-melanogenesis	[28,41,94]
*Ecklonia cava* subsp. *stolonifera*	Anti-allergic effects	[47]
*Sargassum horridum*	Antioxidant	[123,168]
*Sargassum siliquastrum*	Photoprotective effect	[113,121]
*Ecklonia cava*	Laxative effect of phlorotannin	[101]
*Ecklonia cava*	Potential natural muscle-building supplements	[169]
*Ishige okamurae*	Tyrosinase inhibition	[28,170,171]
*Fucus vesiculosus*	Antitumor activity of phlorotannin	[82]
*Sargassum horneri*	Antioxidant	[28,113,172]
*Himanthalia elongata*	Anti-inflammatory properties of phlorotannins	[162]
*Ecklonia cava*	Antiviral activity of phlorotannin	[22]
*Ecklonia stolonifera* Okamura	Tyrosinase inhibition	[7,168]
*Sargassum tenerrimum*	Antibacterial, antioxidant, and anti-inflammatory activities post-treatment.	[111]
*Sargassum fusiforme*	Anti-melanogenesis, downregulated the expression of tyrosinase-1 (TRP-1)	[35,69,113,166]
*Ecklonia cava*	Neuro-protective effects	[3,96]
*Ecklonia bicyclis*	Tyrosinase inhibition	[7,13,28,49,173]
*Ecklonia cava*	Sedative-hypnotics	[174]
*Padina tetrastromatica*	Photoprotective effect	[49,167]

## Data Availability

No new data were created or analyzed in this study. Data sharing is not applicable to this article.

## Abbreviations

The following abbreviations are used in this manuscript:

**Abbreviation**	**Full Term**
ASE	Accelerated Solvent Extraction
DAD	Diode-Array Detection
DW	Dry Weight
EAE	Enzyme-Assisted Extraction
FC	Folin–Ciocalteu Assay
GAE	Gallic Acid Equivalents
HPLC	High-Performance Liquid Chromatography
LCA	Life-Cycle Assessment
LC-MS/MS	Liquid Chromatography–Tandem Mass Spectrometry
MAE	Microwave-Assisted Extraction
NADES	Natural Deep Eutectic Solvents
NMR	Nuclear Magnetic Resonance
ORAC	Oxygen Radical Absorbance Capacity
PGE	Phloroglucinol Equivalents
PLE	Pressurized Liquid Extraction
qNMR	Quantitative Nuclear Magnetic Resonance
RP-HPLC	Reversed-Phase High-Performance Liquid Chromatography
SPE	Solid-Phase Extraction
TPC	Total Phenolic Content
TEA	Techno-Economic Analysis
UAE	Ultrasound-Assisted Extraction
UHPLC-HRMS/MS	Ultra-High-Performance Liquid Chromatography–High-Resolution Tandem Mass Spectrometry
UV–Vis	Ultraviolet–Visible Spectroscopy
WR-NADES	Water-Rich Natural Deep Eutectic Solvents
XAD-7	Amberlite XAD-7 Adsorption Resin
